# Knowledge, Attitude, and Practices Towards Osteoporosis Among Adults in the United Arab Emirates (UAE) in 2023

**DOI:** 10.7759/cureus.56084

**Published:** 2024-03-13

**Authors:** Quds Alani, Massara Yassir, Rafeef Mansoor, Raya Flayyih, Najma Ali Saifuddin Yaqoob, Hessa Rafeeq, Samia Farghaly, Soha Abd El Aziz

**Affiliations:** 1 Research, Dubai Medical College for Girls, Dubai, ARE; 2 Psychology, Dubai Medical College for Girls, Dubai, ARE; 3 Geriatrics, Dubai Health, Dubai, ARE

**Keywords:** bone mass density, practice, attitude, knowledge, bone fracture, osteoporosis

## Abstract

Background: Osteoporosis, the silent epidemic, is defined as a systemic skeletal disease characterized by low mineral bone mass and micro-architectural deterioration of bone tissue. Osteoporosis is considered a burden to global economic, social, and health development.

Osteoporosis exerts a substantial global influence, markedly influencing rates of illness and death on a broad scale. Clinical features of osteoporosis can include chronic back pain, loss of height, and a stooped posture, as well as an increased risk of fractures in the spine, hip, and wrist. Accurate identification and monitoring of these clinical features are essential for effective management and treatment of osteoporosis. This study aims to identify the knowledge, attitudes, and practices (KAP) of adults (over 18 years) about osteoporosis and identify relations between knowledge, attitudes, and practices with demographic data. Furthermore, to assess the risk factors and preventive measures for osteoporosis.

Methodology: Data from 446 responders were collected using a Google Forms questionnaire, including questions to assess knowledge, attitudes, and practice levels among adults 18 years and above in the United Arab Emirates (UAE). The collected data and statistical analysis were done through the IBM® SPSS® Statistics. Chi-Square was used in SPSS Statistics; the chi-square test was used for the relation between categorical variables, and P less than 0.05 was the cut-off level of significance.

Results: The research revealed that 41.9% of the participants had good knowledge, 38.8% had a positive attitude, and 45.3% had poor practices. The results also showed that there is a statistically significant correlation between gender and knowledge, attitudes, and practices.

Conclusion: Our research demonstrates that there’s a statistically significant correlation between gender variables with knowledge, attitudes, and practices. These findings have important implications in assessing the correlation between variables in our research that could be used to prevent osteoporosis further, target the specific demographic group, and provide the required education. Overall, our research contributes to a better understanding of the knowledge, attitude, and practices towards Osteoporosis among adults in the UAE and underscores the importance of further awareness in this area.

## Introduction

Osteoporosis, the silent epidemic, is defined as a systemic skeletal disease characterized by low mineral bone mass and microarchitectural deterioration of bone tissue [1]. Osteoporosis is considered a global economic, social, and health burden. It affects approximately 200 million people globally, significantly impacting morbidity and mortality rates [2].

As stated in the guidelines of the Department of Health in Abu Dhabi (DOH), “If not prevented or left untreated, osteoporosis affects quality-adjusted life years as it can progress into broken bones, also known as fractures". Furthermore, the guidelines highlight that “The changes in quality of life years and disability suffered by people with complications from osteoporosis impact their contribution to an effective and healthy society” [3]. The prevalence of osteoporosis in the UAE was 3.1% (2.7% in men, 3.2% in women) among 3985 Emiratis aged 18-85 years old using quantitative ultrasound (QUS) [4].

Genetic factors play a significant role in determining whether an individual is at increased risk of osteoporosis. However, lifestyle factors such as diet and physical activity also influence bone development in youth and the rate of bone loss later in life (International Osteoporosis Foundation, 2023) [1]. This highlights the importance of assessing the KAP of the population to prevent osteoporosis and its complications through modifiable risk factors.

Osteoporosis is a worldwide disease characterized by reduced bone mass and alteration of bone architecture, resulting in increased bone fragility and fracture risk. Causes of osteoporosis include increasing age, female sex, postmenopausal status, hypogonadism or premature ovarian failure, low body mass index, ethnic background, rheumatoid arthritis, low bone mineral density (BMD), vitamin D deficiency, low calcium intake, hyperkyphosis, current smoking, alcohol abuse, immobilization, and long-term use of certain medications [5,6]. The osteoporosis diagnosis is established by measuring the BMD of the hip and spine using dual-energy X-ray absorptiometry. According to the WHO criteria, osteoporosis is defined as a BMD that lies 2.5 standard deviations or more below the average value for young, healthy women [7]. Bone turnover biomarker detection may be useful in monitoring osteoporosis treatment and assessing fracture risk but not for the diagnosis of osteoporosis. Management of osteoporosis consists of nonpharmacological interventions, which are recommended for all subjects, and pharmacological therapy in all postmenopausal women who have had an osteoporotic fracture or have BMD values consistent with osteoporosis.

## Materials and methods

The study is a descriptive cross-sectional study, aiming to identify if the characteristics of age, gender, educational level, and socioeconomic status correlate with the degree of knowledge, attitudes, and practices toward osteoporosis among the population of the United Arab Emirates; the study was conducted between January 2023 and May 2023.

The target population and inclusion criteria encompass females and males over the age of 18 years residing in the United Arab Emirates, including locals and residents. Exclusion criteria include females and males below the age of 18 years and people who are not residing in the UAE.

The sample size was calculated to be 381 with a 5% degree of precision using the computer program EPIRINFO Version 7, 95% confidence interval, 5%-degree precision, and 45.2% estimated poor knowledge.

A self-administered questionnaire was distributed in electronic format through Google Forum. The questionnaires were obtained from previous research, with permission from the authors [8,9]. It was then translated into English and back to Arabic to ensure a comprehensive and satisfactory questionnaire. To ensure the validity and reliability of the study, the final translated questionnaire was field tested on a pilot sample of 20 students to address any ambiguity and reliability of the questions. Furthermore, feedback was obtained from experts in the field regarding the design of the study. The questionnaire comprised 4 parts: Sociodemographic variables, knowledge, attitude, and practices toward osteoporosis. The sociodemographic variables included age, gender, nationality, educational level, and income sufficiency.

The assessment of knowledge towards osteoporosis included 10 questions, and responses included yes, no, and I don't know. Yes, was scored as 1. No was scored as 0. I don't know was scored as 0. Knowledge level was divided as follows: Poor knowledge level is 50% or less. The average knowledge level is 50% to less than 75%. A good knowledge level is 75% or more.

Assessment of attitude towards osteoporosis included 6 questions. Responses included yes, no, and I don't know. Yes, it was scored as 3. No was scored as 1. I don't know was scored as 2. Attitude level was divided as follows: Poor attitude level is 50% or less. The average attitude level is 50% to less than 75%. A good attitude level is 75% or more.

Assessment of practice towards osteoporosis included 9 questions. Responses included yes and no. Yes, it was scored as 2. No was scored as 1. The practice level was divided as follows: Poor practice level is 50% or less. The average practice level is 50% to less than 75%. The good practice level is 75% or more.

Data was entered into an Excel sheet. The statistical analysis of the data was done through the IBM® SPSS® Statistics version 29. Chi-square was used in SPSS Statistics; the chi-square test was used for the relation between categorical variables, and P less than 0.05 was the cut-off level of significance.

The proposal was submitted to the Dubai Medical College for Girls (DMCG) Research Review Board to obtain approval from the ethics board. The participants were informed of the aim of the study. Informed and written consent was taken from all participants. Anonymity and confidentiality of the information was maintained. Analysis was done in a manner that ensured there was no connection between participants and the result. It was ensured that no physical, mental, or social stress was caused to the participants or any person through this research.

## Results

The total number of participants that met the inclusion criteria was 446 participants. A comprehensive examination of the participants' sociodemographic data showed that within this cohort (N = 309), 69.28% identified as female, while (N = 137) 30.7% identified as male. Age distribution revealed a notable representation of younger individuals, with (N = 199) 44.6% falling within the 18 to 24-year bracket. Subsequently, (N = 119) 26.7% were between 40 and 54 years, indicating a varied age spread. Nationality-wise, the majority (N = 394), 88.34% of participants, were non-local, contrasting with (N = 52 ), 11.66%, who were local residents. Regarding educational attainment, a significant proportion of participants exhibited high levels of education, with (N=277) 62.11% holding Bachelor's degrees and (N = 65) 15.92% possessing post-graduate qualifications. However, a smaller fraction had completed only primary education (N = 2) 0.45%, and (N = 102) 22.87% were high school graduates. Regarding income, (N = 73) 16.4% of participants reported having insufficient income, while (N = 155) 34.8% indicated their income was sufficient. Interestingly, (N = 79) 17.7% reported having sufficient income with savings, suggesting a degree of financial stability. Notably, (N = 139) 31.2% of participants did not provide income data, indicating potential variability or uncertainty in income sources among this subset (Table 1).

**Table 1:** Sociodemographic data of participants Total Number of Participants: 446 Frequency represents the number of participants in each variable category. * Missing: participants with no income/no stable income.

Sociodemographic data of participants			
Variable		Frequency	Percentage (%)
Gender	Female	309	69.28%
	Male	137	30.72%
Age	> 55 years	30	6.7%
	18 - 24 years	199	44.6%
	25 - 39 years	98	22.0%
	40 - 54 years	119	26.7%
Nationality	Local	52	11.66%
	Non-local	394	88.34%
Educational Level	Primary Education	2	0.45%
	High school Graduate	102	22.87%
	Bachelor’s Degree	277	62.11%
	Post-Graduate Degree	65	15.92%
Income	Not Sufficient	73	16.4%
	Sufficient	155	34.8%
	Sufficient and saving	79	17.7%
	Missing*	139	31.2%

Knowledge level

Based on the research findings, the knowledge levels of the 446 participants regarding osteoporosis were assessed. The results indicate that (N=187) 41.9% of the participants demonstrated a good understanding of osteoporosis. However, a considerable proportion, (N=176) 39.5%, exhibited a poor knowledge level about the condition. Additionally, (N=83) 18.6% of the participants had an average knowledge level (Table 2).

**Table 2:** Knowledge level N = Number of Participants

Knowledge Level		Frequency (N)	Percent
Valid	Poor	176	39.5%
	Average	83	18.6%
	Good	187	41.9%
	Total	446	100.0%

Upon cross-tabulation of knowledge levels with gender, it was observed that among the 309 female participants, nearly half (N = 151), 48.9%, exhibited good knowledge about osteoporosis. However, a significant portion, (N=97) 31.4%, displayed a poor knowledge level about the condition. In contrast, among the 137 male participants, only (N=36) 26.3% demonstrated a good knowledge level, while a substantial majority, (N=79) 57.7%, exhibited a poor knowledge level (Table 3). The chi-square tests were conducted to explore the relationship between knowledge level and gender in the research study. The results indicate a statistically significant association between these variables across all metrics, including the Pearson chi-square, likelihood ratio, and Fisher-Freeman-Halton Exact test (p < 0.001). This suggests that gender and knowledge level are not independent of each other within the dataset. In other words, there appears to be a relationship between gender and the level of knowledge exhibited by the participants (Table 4).

**Table 3:** Knowledge based on Gender Count (N) = Number of participants.

Variable			Knowledge Level		
			Poor	Average	Good
Gender	Female	Count (N)	97	61	151
		% within Gender	31.4%	19.7%	48.9%
	Male	Count (N)	79	22	36
		% within Gender	57.7%	16.1%	26.3%
Total		Count (N)	176	83	187
		% within Gender	39.5%	18.6%	41.9%

**Table 4:** Chi-square tests of knowledge and gender A p-value of 0.05 and below was considered statistically significant. a. 0 cells (0.0%) have an expected count of less than 5. The minimum expected count is 25.50. b. Based on 10000 sampled tables with starting seed 2000000.

Chi-square Tests						
Test	Value	df	Asymptotic Significance (2-sided)	Monte Carlo Sig. (2-sided)		
				Significance	95% Confidence Interval	
					Lower Bound	Upper Bound
Pearson Chi-square	28.847^a^	2	0.000	<0.001^b^	0.000	0.000
Likelihood Ratio	28.863	2	0.000	<0.001^b^	0.000	0.000
Fisher-Freeman-Halton Exact Test	28.626	-	-	<0.001^b^	0.000	0.000

Number of Valid Cases	446

A cross-tabulation reveals varying levels of knowledge across different age groups. Among participants above 55 years old, a notable majority, (N = 16) 53.3%, demonstrated a good knowledge level, while (N = 13) 43.3% within the same age group exhibited poor knowledge. In contrast, participants in the age group of 18 to 24 years old displayed a distribution of (N = 68) 34.2% with poor knowledge and (N = 94) 47.2% with good knowledge levels. Similarly, individuals aged 25 to 39 showed a prevalence of (N=41) 41.8% with poor knowledge and (N = 38) 38.8% with good knowledge. Furthermore, those aged 40 to 54 years old had (N = 54) 45.4% exhibiting poor knowledge and (N = 39) 32.8% displaying good knowledge levels (Table 5). When correlating knowledge level with age, the calculated p-value was 0.061 in the Pearson chi-square test, indicating statistical insignificance.

**Table 5:** Knowledge level based on Age. Count (N) = Number of participants.

Variable			Knowledge Level		
			Poor	Average	Good
Age	> 55 years	Count (N)	13	1	16
		% within Age	43.3%	3.3%	53.3%
	18 - 24 years	Count (N)	68	37	94
		% within Age	34.2%	18.6%	47.2%
	25 - 39 years	Count (N)	41	19	38
		% within Age	41.8%	19.4%	38.8%
	40 - 54 years	Count (N)	54	26	39
		% within Age	45.4%	21.8%	32.8%
Total		Count (N)	176	83	187
		% within Age	39.5%	18.6%	41.9%

The research findings on the knowledge levels regarding osteoporosis include an examination of participants' nationality-local Emirati or non-local (non-Emirati). Upon cross-tabulation, it was observed that among the 52 local Emirati participants, a substantial proportion, (N = 20) 38.5%, exhibited a good understanding of osteoporosis, while (N = 19) 36.5% displayed poor knowledge. Conversely, among the 394 non-local participants, a slightly higher percentage, (N = 167) 42.4%, demonstrated a good knowledge level, with (N = 157) 39.8% showing poor knowledge (Table 6). When correlating knowledge level with nationality, the calculated p-value was 0.452 in the Pearson chi-square test, indicating statistical insignificance.

**Table 6:** Knowledge level based on nationality Count (N) = Number of participants Local: Emirati Non-local: Not Emirati

Variable			Knowledge Level		
			Poor	Average	Good
Nationality	Local	Count (N)	19	13	20
		% within Nationality	36.5%	25.0%	38.5%
	Non-local	Count (N)	157	70	167
		% within Nationality	39.8%	17.8%	42.4%
Total		Count (N)	176	83	187
		% within Nationality	39.5%	18.6%	41.9%

Moreover, there is a cross-tabulation of knowledge levels with their educational backgrounds. Participants were categorized into different educational levels, including Bachelor's Degree, High school graduate, Postgraduate Degree, and Primary education. Among participants with a Bachelor's Degree (N=277), the majority (N= 122), 44.0%, demonstrated a good knowledge level, while (N= 100) 36.1% exhibited a poor knowledge level. Similarly, among High school graduates (N=102), (N= 40) 39.2% displayed a good knowledge level, while (N= 46) 45.1% had a poor knowledge level. In contrast, participants with a Postgraduate Degree (N=65) showed a slightly different pattern, with (N= 24) 36.9% demonstrating a good knowledge level and (N= 29) 44.6% exhibiting a poor knowledge level. Interestingly, only a small number of participants had a Primary education (N=2), and they showed an equal split between good and poor knowledge levels (Table 7). When correlating knowledge level with educational level, the calculated p-value was 0.693 in the Pearson chi-square test, suggesting statistical insignificance.

**Table 7:** Knowledge level based on educational level Count (N) = Number of participants.

Variable			Knowledge Level		
			Poor	Average	Good
Educational level	Bachelor's Degree	Count (N)	100	55	122
		% within Educational level	36.1%	19.9%	44.0%
	High school graduate	Count (N)	46	16	40
		% within Educational level	45.1%	15.7%	39.2%
	Postgraduate Degree	Count (N)	29	12	24
		% within Educational level	44.6%	18.5%	36.9%
	Primary	Count (N)	1	0	1
		% within Educational level	50.0%	0.0%	50.0%
Total		Count (N)	176	83	187
		% within Educational level	39.5%	18.6%	41.9%

When analyzing the knowledge levels with their income levels, participants were classified into three income categories: "Not Sufficient," "Sufficient," and "Sufficient and saving". Among participants with "Not Sufficient" income, the majority (N=28), 38.4%, demonstrated a good knowledge level, while (N=35)47.9% exhibited a poor knowledge level. Similarly, among those with "Sufficient" income, (N=67) 43.2% showed a good knowledge level, with (N=57) 36.8% displaying a poor knowledge level. For participants with "Sufficient and saving" income, there was a slightly different pattern, with (N=25) 31.6% demonstrating a good knowledge level and (N=36) 45.6% exhibiting a poor knowledge level. When correlating knowledge level with income level, the calculated p-value was 0.248 in the Pearson chi-square test, indicating statistical insignificance (Table 8).

**Table 8:** Knowledge based on income Count (N): Number of participants

Variable			Knowledge Level		
			poor	average	good
Income	Not Sufficient	Count (N)	35	10	28
		% within Income	47.9%	13.7%	38.4%
	Sufficient	Count (N)	57	31	67
		% within Income	36.8%	20.0%	43.2%
	Sufficient and saving	Count (N)	36	18	25
		% within Income	45.6%	22.8%	31.6%
Total		Count	128	59	120
		% within Income	41.7%	19.2%	39.1%

Attitudes

The research findings present a comprehensive analysis of the attitude levels of 446 participants toward osteoporosis. The participants' attitudes were categorized into three levels: negative, neutral, and positive. Among the participants, (N=173) 38.8% expressed a negative attitude towards osteoporosis. In contrast, an equal percentage of participants (N=173), 38.8%, held a positive attitude towards the condition. Additionally, (N=100) 22.4% of participants maintained neutral attitudes regarding osteoporosis (Table 9).

**Table 9:** Attitudes Frequency (N) = Number of participants.

Attitude		Frequency (N)	Percent
Valid	Negative	173	38.8%
	Neutral	100	22.4%
	Positive	173	38.8%
	Total	446	100.0

Among female participants, (N=117) 37.9% expressed a negative attitude towards osteoporosis, while (N=113) 36.6% held a positive attitude. Additionally, (N=79) 25.6% maintained a neutral stance regarding the condition. Similarly, among male participants, (N=56) 40.9% demonstrated a negative attitude, while (N=60) 43.8% held a positive attitude towards osteoporosis. Only (N=21) 15.3% of male participants expressed a neutral attitude (Table 10). The chi-square tests were conducted to explore the relationship between attitude levels and gender within the research study. The results indicate a marginally significant association between these variables, as evidenced by the p-values of 0.051 for the Pearson chi-square test and 0.044 for the Likelihood Ratio test. While these p-values are slightly above the conventional threshold of 0.05 for statistical significance, they still suggest a potential relationship between attitude levels and gender. The Fisher-Freeman-Halton Exact test also yielded a significance level of 0.050. This indicates that, although not statistically significant at the conventional threshold, there may still be a meaningful association between attitude levels and gender within the dataset. Further examination could involve investigating whether specific gender groups exhibit different attitude levels and exploring the nuances of this relationship. With a dataset comprising 446 valid cases, these findings provide valuable insights into the interplay between gender and attitudes within the research context (Table 11).

**Table 10:** Attitudes based on gender Count (N) = Number of participants.

Variable			Attitude		
			Negative	Neutral	Positive
Gender	Female	Count (N)	117	79	113
		% within Gender	37.9%	25.6%	36.6%
	Male	Count (N)	56	21	60
		% within Gender	40.9%	15.3%	43.8%
Total		Count (N)	173	100	173
		% within Gender	38.8%	22.4%	38.8%

**Table 11:** Chi-square tests of attitudes and gender A p-value of 0.05 and below was considered statistically significant. a. 0 cells (0.0%) have an expected count of less than 5. The minimum expected count is 30.72. b. Based on 10000 sampled tables with starting seed 1502173562.

Chi-Square Tests						
Test	Value	df	Asymptotic Significance (2-sided)	Monte Carlo Sig. (2-sided)		
				Significance	95% Confidence Interval	
					Lower Bound	Upper Bound
Pearson Chi-Square	5.937^a^	2	0.051	0.051^b^	0.047	0.055
Likelihood Ratio	6.235	2	0.044	0.045^b^	0.041	0.049
Fisher-Freeman-Halton Exact Test	6.090	-	-	0.050^b^	0.046	0.054

Number of Valid Cases	446

Among participants over 55 years, (N=7) 23.3% expressed a negative attitude towards osteoporosis, while (N=13) 43.3% held a positive attitude. For participants aged between 18 to 24 years, (N=79) 39.7% demonstrated a negative attitude, while (N=70) 35.2% held a positive attitude towards osteoporosis. Similarly, among participants aged between 25 to 39 years, (N=41) 41.8% exhibited a negative attitude, while (N=38) 38.8% expressed a positive attitude towards osteoporosis. For participants aged between 40 to 54 years, (N=46) 38.7% demonstrated a negative attitude, while (N=52) 43.7% held a positive attitude towards osteoporosis. The cross-tabulation of attitude level with age in chi-square tests yielded a p-value of 0.278 in the Pearson chi-square test, indicating that the correlation was statistically insignificant (Table 12).

**Table 12:** Attitudes based on age Count (N) = Number of participants.

Variable			Attitudes		
			Negative	Neutral	Positive
Age	> 55 years	Count (N)	7	10	13
		% within Age	23.3%	33.3%	43.3%
	18 - 24 years	Count (N)	79	50	70
		% within Age	39.7%	25.1%	35.2%
	25 - 39 years	Count (N)	41	19	38
		% within Age	41.8%	19.4%	38.8%
	40 - 54 years	Count (N)	46	21	52
		% within Age	38.7%	17.6%	43.7%
Total		Count (N)	173	100	173
		% within Age	38.8%	22.4%	38.8%

Among local participants, (N=20) 38.5% expressed a negative attitude towards osteoporosis, while (N=17) 32.7% held a positive attitude. Similarly, among non-local participants, (N=153) 38.8% demonstrated a negative attitude, while (N=156) 39.6% held a positive attitude towards osteoporosis. Meanwhile, (N=85) 21.6% expressed a neutral attitude. The correlation between attitude level and nationality resulted in a p-value of 0.438 in the Pearson chi-square test, suggesting statistical insignificance (Table 13).

**Table 13:** Attitudes based on nationality Count (N) = Number of participants. Local: Emirati Non-local: Not Emirati

Variable			Attitude Level		
			Negative	Neutral	Positive
Nationality	Local	Count (N)	20	15	17
		% within Nationality	38.5%	28.8%	32.7%
	Non-local	Count (N)	153	85	156
		% within Nationality	38.8%	21.6%	39.6%
Total		Count (N)	173	100	173
		% within Nationality	38.8%	22.4%	38.8%

The findings unveil a nuanced panorama of attitudes among the participants towards osteoporosis, cross-tabulated with their educational backgrounds. Participants holding a Bachelor's Degree (N=115), 41.5%, displayed a positive attitude towards osteoporosis, while (N=97) 35.0% harbored a negative outlook. For participants who graduated high school, (N=37) 36.3% exhibited a positive attitude, while (N=44) 43.1% held a negative perspective towards osteoporosis. Among participants with a Postgraduate Degree, (N=19) 29.2% showcased a positive attitude, while (N=32) 49.2% adhered to a negative viewpoint towards osteoporosis. Notably, participants with a primary education level (total count: 2) displayed a unanimous positive attitude towards osteoporosis. The analysis of attitude level in relation to the educational level produced a p-value of 0.161 in the Pearson chi-square test, indicating statistical insignificance (Table 14).

**Table 14:** Attitudes based on educational level Count (N): Number of participants

Variable			Attitudes		
			Negative	Neutral	Positive
Educational level	Bachelor's Degree	Count (N)	97	65	115
		% within Educational level	35.0%	23.5%	41.5%
	High school graduate	Count (N)	44	21	37
		% within Educational level	43.1%	20.6%	36.3%
	Postgraduate Degree	Count (N)	32	14	19
		% within Educational level	49.2%	21.5%	29.2%
	Primary	Count (N)	0	0	2
		% within Educational level	0.0%	0.0%	100.0%
Total		Count (N)	173	100	173
		% within Educational level	38.8%	22.4%	38.8%

Participants with insufficient income showcased a positive attitude towards osteoporosis in (N=34) 46.6% of cases, while (N=28) 38.4% exhibited a negative outlook. Within the cohort of individuals possessing sufficient income, (N=69) 44.5% displayed a positive attitude, whereas (N=52) 33.5% held a negative attitude. Among participants with sufficient income and savings, (N=30) 38.0% displayed a positive attitude, with (N=32) 40.5% adhering to a negative viewpoint. When examining the correlation between attitude level and income level, the obtained p-value was 0.594 in the Pearson chi-square test, signifying statistical insignificance (Table 15).

**Table 15:** Attitudes based on income Count (N): Number of participants

Variable			Attitudes		
			Negative	Neutral	Positive
Income	Not Sufficient	Count (N)	28	11	34
		% within Income	38.4%	15.1%	46.6%
	Sufficient	Count (N)	52	34	69
		% within Income	33.5%	21.9%	44.5%
	Sufficient and saving	Count (N)	32	17	30
		% within Income	40.5%	21.5%	38.0%
Total		Count (N)	112	62	133
		% within Income	36.5%	20.2%	43.3%

Practices

The research findings shed light on the practices towards osteoporosis among 446 participants. Participants' practices were categorized into three levels: poor, average, and good. Among the participants, (N=202) 45.3% demonstrated poor practices towards osteoporosis. Additionally, (N=112) 25.1% of participants exhibited average practices, while (N=132) 29.6% displayed good practices towards the condition (Table 16).

**Table 16:** Practice levels Frequency (N) = Number of participants

Practices level		Frequency (N)	Percent (%)
Valid	Poor	202	45.3%
	Average	112	25.1%
	Good	132	29.6%
	Total	446	100.0%

Analysis of gender in conjunction with practices towards osteoporosis delineates distinct trends within the participant cohort. Female participants show a higher prevalence of poor practices (N=149) 48.2% compared to males (N=53) 38.7%. Conversely, a greater percentage of males exhibit good practices (N=58), 42.3%, in contrast to females (N=74), 23.9% (Table 17). The chi-square tests were conducted to examine the relationship between gender and practices towards osteoporosis within the research study. The results indicate a highly significant association between these variables, with all test metrics, including the Pearson chi-square, likelihood ratio, and Fisher-Freeman-Halton Exact test, yielding p-values of 0.000. This suggests that gender significantly influences the observed practices of participants related to osteoporosis, with the probability of this association occurring by chance being extremely low. The 95% confidence intervals for the significance levels consistently fall below 0.001, indicating the robustness of these findings. With a dataset comprising 446 valid cases and a minimum expected count of 34.40, these results underscore the substantive connection between gender and practices targeted at osteoporosis (Table 18).

**Table 17:** Practices based on gender Count (N) = Number of participants

Variable			Practices		
			Poor	Average	Good
Gender	Female	Count (N)	149	86	74
		% within Gender	48.2%	27.8%	23.9%
	Male	Count (N)	53	26	58
		% within Gender	38.7%	19.0%	42.3%
Total		Count (N)	202	112	132
		% within Gender	45.3%	25.1%	29.6%

**Table 18:** Chi-square tests of practices and gender p-value is considered statistically significant at p<0.05. a. 0 cells (0.0%) have an expected count of less than 5. The minimum expected count is 34.40. b. Based on 10000 sampled tables with starting seed 215962969.

Chi-Square Tests						
Test	Value	df	Asymptotic Significance (2-sided)	Monte Carlo Sig. (2-sided)		
				Significance	95% Confidence Interval	
					Lower Bound	Upper Bound
Pearson Chi-Square	15.711^a^	2	0.000	<0.001^b^	0.000	0.000
Likelihood Ratio	15.270	2	0.000	<0.001^b^	0.000	0.001
Fisher-Freeman-Halton Exact Test	15.157	-	-	<0.001^b^	0.000	0.001

Number of Valid Cases	446

The cross-tabulation of age with practices towards osteoporosis reveals noteworthy patterns. Individuals aged over 55 years exhibit a significantly higher proportion of good practices (N=15), 50.0%, while the majority of those aged between 18 to 24 years demonstrate poor practices (N=96), 48.2%. Similarly, participants aged between 25 to 39 years and 40 to 54 years also display substantial percentages of poor practices (N=46) 46.9% and (N=52) 43.7%, respectively). The cross-tabulation of practices with age resulted in a p-value of 0.142 in the Pearson Chi-square test, indicating statistical insignificance (Table 19).

**Table 19:** Practices based on age Count (N): Number of participants

Variable			Practices		
			Poor	Average	Good
AGE	> 55 years	Count (N)	8	7	15
		% within AGE	26.7%	23.3%	50.0%
	18 - 24 years	Count (N)	96	54	49
		% within AGE	48.2%	27.1%	24.6%
	25 - 39 years	Count (N)	46	22	30
		% within AGE	46.9%	22.4%	30.6%
	40 - 54 years	Count (N)	52	29	38
		% within AGE	43.7%	24.4%	31.9%
Total		Count (N)	202	112	132
		% within AGE	45.3%	25.1%	29.6%

The cross-tabulation of nationality with practices towards osteoporosis highlights notable variations among participants. While both local and non-local participants exhibit similar percentages of poor practices (N=24) 46.2% and (N=178) 45.2%, respectively), there is a noticeable difference in the prevalence of good practices, with non-local participants showing a higher percentage (N=118) 29.9% compared to local participants (N=14) 26.9%. These findings suggest potential cultural or contextual factors influencing osteoporosis-related practices among different nationalities, warranting further investigation. In cross-tabulating practices with nationality, the calculated p-value was 0.892 in the Pearson chi-square test, indicating statistical insignificance (Table 20).

**Table 20:** Practices based on nationality Count (N) = Number of participants. Local: Emirati Non-local: Not Emirati

Variable			Practice		
			Poor	Average	Good
Nationality	Local	Count (N)	24	14	14
		% within Nationality	46.2%	26.9%	26.9%
	Non-local	Count (N)	178	98	118
		% within Nationality	45.2%	24.9%	29.9%
Total		Count (N)	202	112	132
		% within Nationality	45.3%	25.1%	29.6%

Examination of educational level alongside practices towards osteoporosis unveils intriguing insights among the participant cohort. Participants with a postgraduate degree demonstrate the highest proportion of poor practices (N=33), 50.8%, while those with a bachelor's degree exhibit the highest percentage of good practices (N=73), 26.4%. Moreover, individuals with a primary education level display a stark contrast, with a 100% rate of good practices, albeit within a small sample size (N=2). These findings underscore the influence of educational attainment on osteoporosis-related practices and highlight the need for tailored educational interventions across different educational backgrounds. The cross-tabulation of practices with educational level yielded a p-value of 0.255 in the Pearson Chi-square test, suggesting statistical insignificance (Table 21)

**Table 21:** Practices based on educational level Count (N): Number of participants

Variable			Practices		
			Poor	Average	Good
Educational level	Bachelor's Degree	Count (N)	128	76	73
		% within Educational level	46.2%	27.4%	26.4%
	High school graduate	Count (N)	41	23	38
		% within Educational level	40.2%	22.5%	37.3%
	Postgraduate Degree	Count (N)	33	12	20
		% within Educational level	50.8%	18.5%	30.8%
	Primary	Count (N)	0	1	1
		% within Educational level	0.0%	50.0%	50.0%
Total		Count (N)	202	112	132
		% within Educational level	45.3%	25.1%	29.6%

The cross-tabulation of income level with practices towards osteoporosis elucidates distinct patterns among participants. While individuals with sufficient income and savings demonstrate the highest percentage of good practices (N=33) 41.8%, those with insufficient income exhibit a higher prevalence of poor practices (N=32) 43.8%. Interestingly, participants with sufficient income also display a notable percentage of poor practices (N=77) 49.7%. These findings underscore the influence of socioeconomic factors on osteoporosis-related practices, emphasizing the importance of equitable access to resources and targeted interventions to address barriers to optimal practices across diverse income levels. The correlation between practices and income level resulted in a p-value of 0.137 in the Pearson Chi-square test, indicating statistical insignificance (Table 22).

**Table 22:** Practices based on income Count (N) = Number of participants.

Variable			Practices		
			Poor	Average	Good
Income	Not Sufficient	Count (N)	32	18	23
		% within Income	43.8%	24.7%	31.5%
	Sufficient	Count (N)	77	36	42
		% within Income	49.7%	23.2%	27.1%
	Sufficient and saving	Count (N)	26	20	33
		% within Income	32.9%	25.3%	41.8%
Total		Count (N)	135	74	98
		% within Income	44.0%	24.1%	31.9%

## Discussion

This study aimed to assess the knowledge, attitudes, and practices (KAP) regarding osteoporosis among 446 participants meeting the inclusion criteria. The socio-demographic profile revealed a predominantly young (18-24 years), female, and non-local participant population, with a majority holding a bachelor's degree and reporting sufficient income levels (Table 1).

Knowledge Level

Analysis of knowledge levels unveiled a mixed understanding of osteoporosis, with significant proportions exhibiting poor knowledge. While 177 participants (41.9%) demonstrated good knowledge, 187 participants (39.5%) displayed poor knowledge. Gender showed statistically significant associations with knowledge levels, with female participants (N=151) (48.9%) and male participants (N=36) (26.3%) exhibiting good knowledge. However, age, nationality, educational level, and income level did not show statistically significant associations with knowledge levels.

Attitudes

Attitudes towards osteoporosis were varied, with a notable percentage expressing negative views. Of the participants, 173 (38.8%) held a positive attitude, while an equal percentage expressed a negative attitude. Gender was a statistically significant predictor of attitudes, with 117 male participants (43.8%) holding positive attitudes. However, nationality, educational level, and income level did not show statistically significant associations with attitudes.

Practices

Participants demonstrated varying levels of practices towards osteoporosis, with a considerable proportion exhibiting poor practices. Of the participants, 202 (45.3%) demonstrated poor practices. Gender had a statistically significant association with practices, with 150 female participants (48.2%) engaging in poor practices. However, age, nationality, educational level, and income level did not show statistically significant associations with practices.

These findings underscore the need for targeted interventions to improve knowledge, attitudes, and practices regarding osteoporosis. Educational campaigns tailored to address misconceptions and promote healthy behaviors should be implemented. Additionally, interventions targeting specific sociodemographic groups, such as those with lower educational attainment or income levels, may be warranted to address disparities in KAP levels.

In comparing our study's findings on osteoporosis awareness, attitudes, and practices with a 2018 study conducted in the UAE titled "Assessment of knowledge, attitude, and practice (KAP) of osteoporosis and its predictors among university students: a cross-sectional study, UAE" [10], a noteworthy consistency emerges. Both studies identified a statistically significant correlation between gender and osteoporosis-related knowledge, attitude, and practice among university students. The 2018 study revealed an average KAP score of 74% for female students compared to 65% for male students. Similarly, our study demonstrated a significant association between gender and knowledge levels, with female participants exhibiting a higher proportion of good knowledge. Importantly, neither study found statistically significant results in the correlation of KAP with other variables.

A study conducted in Riyadh in 2019 examined the knowledge, attitudes, and practices regarding osteoporosis among young adults [11]. The findings revealed a notable deficiency in knowledge across the overall population surveyed. However, a distinct pattern emerged when examining different age groups. Among these, individuals aged 21-25 exhibited the highest level of knowledge concerning osteoporosis, contrasting with the general trend of low awareness. Interestingly, our research findings diverged from this pattern. Our results showed that participants aged 55 years old and above displayed the most profound understanding of osteoporosis among all age groups involved in the study. This discrepancy underscores the importance of considering diverse demographic factors when assessing knowledge levels and attitudes toward osteoporosis within a population. Another notable finding emerged: no discernible difference was observed between the male and female gender groups. However, our own research yielded contrasting results. Upon comparing our findings, we found a statistically significant difference between the two genders, which was evident across all categories: knowledge, attitudes, and practices. Specifically, females demonstrated higher levels of knowledge regarding osteoporosis compared to males. Conversely, males exhibited superior attitudes and practices related to osteoporosis prevention and management. This discrepancy underscores the importance of recognizing gender-specific nuances when devising educational and interventional strategies aimed at enhancing osteoporosis awareness and prevention among populations.

In the study examining the knowledge, attitude, and practice of osteoporosis among adult patients in Bashair Hospital, Sudan, in 2021 [12], a significant finding emerged: factors such as age, level of education, and gender exerted a notable influence on the levels of knowledge, attitude, and practice regarding osteoporosis. However, in contrast to these findings, our own research revealed statistically significant results only within genders. This suggests that while gender played a significant role in shaping knowledge, attitudes, and practices related to osteoporosis, other demographic factors such as age and level of education did not show significant associations in our study. These differing results highlight the importance of considering contextual factors and variations in study populations when interpreting osteoporosis awareness and management findings.

In the investigation into the knowledge, attitude, and practice of osteoporosis among adult women in Majmmah City, Saudi Arabia [13], a noteworthy finding emerged: while younger populations exhibited higher levels of knowledge and attitude towards osteoporosis, the middle-aged population demonstrated better preventive measures. However, our research produced somewhat different results. Contrary to the findings in Majmmah City, participants aged 55 years and above in our study displayed the highest knowledge levels among all age groups. Additionally, regarding attitudes, the age group of 40-54 years scored the highest. Interestingly, participants aged 55 years and above in our research showed the highest scores regarding preventive measures or practices. These differences underscore the need to consider regional and demographic variations when evaluating and dealing with strategies related to the awareness and management of osteoporosis.

In contrast to our study examining osteoporosis knowledge, attitudes, and practices (KAP) in the general adult population in the UAE, the research published in the Iraqi National Journal of Medicine [14] focused on family physicians in the Babylon governate. Notably, the latter study revealed that among female physicians, 4.6% demonstrated good knowledge, 88.9% exhibited positive attitudes, and 32.2% displayed good practices. Among male physicians, 22.3% had good knowledge, 74.7% showed positive attitudes, and 88.9% had good practices. Conversely, our study found that, among adults in the UAE, 41.9% had good knowledge, 38.8% displayed positive attitudes, and 29.6% showed good practices. Among family physicians, only 6% had good knowledge, while 59% had moderate knowledge, 76% exhibited positive attitudes, and 33% had good practices. These findings suggest a potential disparity in osteoporosis KAP levels between the general adult population and healthcare providers, with physicians in the Babylon governate demonstrating higher knowledge, attitudes, and practices. This disparity may be attributed to differences in educational backgrounds and underscores the importance of tailored interventions to address osteoporosis awareness and practices in distinct demographic groups.

Several limitations are worth noting in our study. Sampling bias could have impacted the generalizability of findings due to the potential overrepresentation or underrepresentation of certain demographic or geographic groups. Recall bias might have affected the accuracy of reported knowledge, attitudes, and practices related to osteoporosis. In addition to the type of our study, a cross-sectional design restricts the establishment of causal relationships or the tracking of changes over time. Additionally, language barriers could have hindered participants' understanding of questions as our questionnaire was only available in Arabic and English; considering the multicultural nature of the UAE with diverse nationalities, this language limitation is noteworthy. These considerations emphasize the need for cautious interpretation and suggest areas for improvement in future research.

## Conclusions

In conclusion, this research successfully met its objectives by assessing adults' knowledge, attitudes, and practices (KAP) towards osteoporosis. The findings revealed less than half of the participants demonstrated good knowledge about osteoporosis, indicating a substantial gap in understanding. Similarly, positive attitudes towards osteoporosis were observed in only a significant minority of participants, highlighting a need for attitude enhancement interventions. Moreover, the study found that merely one-third of participants exhibited good practices concerning osteoporosis prevention and management, indicating significant room for improvement in behavioral aspects. Notably, the analysis unveiled that the sociodemographic factors, except for age groups, did not show statistically significant associations with KAP levels. This suggests that interventions aiming to improve KAP regarding osteoporosis may need to target specific groups.

## Appendices

**Table 23:** Research Questionnaire Four sections of the questionnaire : 1 Sociodemographics 2 Knowledge Level 3 Attitudes 4 Practices

Sociodemographic Data
Age
18 - 24 years
25 - 39 years
40 - 54 years
> 55 years
Gender
Female
Male
Nationality
Local
Non-local
Educational level
Primary
High school graduate
Bachelor’s Degree
Masters
PhD
Income
Not Sufficient
Sufficient
Sufficient and saving
No income

Knowledge Level
What is the correct definition for osteoporosis?
Bone fracture
Loss of bone strength and density
I don’t know
Do you believe osteoporosis increases the risk of bone fractures?
Yes
No
I don't know
Do you believe osteoporosis causes symptoms such as back pain before fractures occur?
Yes
No
I don’t know
Do you believe obtaining a high bone mass during childhood gives protection against the occurrence of osteoporosis in the future?
Yes
No
I don’t know
Who do you think is more prone to osteoporosis?
Women
Men
Children
I don’t know
Do you believe women with white skin are more prone to osteoporosis than others?
Yes
No
I don’t know
Do you think old people are more prone to osteoporosis?
Yes
No
I don’t know
Do you believe women aged 50 and above are more susceptible to fractures at least once before death?
Yes
No
I don’t know
Do you believe that family history of osteoporosis predisposes individuals to osteoporosis?
Yes
No
I don’t know
Do you believe thyroid diseases may predispose to osteoporosis?
Yes
No
I don’t know

Attitudes
Do you believe exposure to falls is an important cause of osteoporosis?
Yes
No
I don’t know
Do you believe that any physical activity is useful for osteoporosis prevention?
Yes
No
I don’t know
Do you believe that drinking two cups of milk daily protects you against osteoporosis?
Yes
No
I don’t know
Do you believe that drinking energy drinks may be a cause of osteoporosis?
Yes
No
I don’t know
Do you believe that smoking contributes to osteoporosis occurrence?
Yes
No
I don’t know
Do you believe weightlifting and practising vigorous activities contribute to an increased chance of osteoporosis?
Yes
No
I don’t know

Practices
Do you eat vegetables as a part of your daily diet?
Yes
No
Do you eat fruits as a part of your daily diet?
Yes
No
Do you take a vitamin D supplement?
Yes
No
Do you drink milk?
Yes
No
Do you follow a balanced diet?
Yes
No
Do you smoke?
Yes
No
Do you consume alcohol?
Yes
No
Do you spend more than 15 minutes in sun daily?
Yes
No
Do you practice physical activity for more than 20 minutes daily?
Yes
No
